# Supplementary material for: Patterns of Intron Gain and Loss in Fungi
Source: PLoS Biol. 2004 Nov 30;2(12):e422. doi: 10.1371/journal.pbio.0020422 (PMC532390; doi:10.1371/journal.pbio.0020422)
Supplement: Table S1 — Also available at http://genes.mit.edu/NielsenEtAl/. (4.3 MB ZIP). [file pbio.0020422.st001.zip › NielsenEtAl/html/1375.html]

AN0828.1.NCU04092.1.MG11078.1.FG03177.1


```
 CLUSTAL W (1.82) Multiple Sequence Alignments - Introns Inserted


Sequence 1: NCU04092.1	625 aa
Sequence 2: MG11078.1	615 aa
Sequence 3: FG03177.1	626 aa
Sequence 4: AN0828.1	619 aa
Alignment Length: 665 aa
Number Identitical Residues: 179 aa
Alignment Score (without introns) 10614


MG11078.1 	---MSGVAG-ISTT------PTQLTG1HDGRLFQKRPG2---------------~MENLR
NCU04092.1	---MSQKTGSVSQSKRLLNYPDPLRG2PDVPYVVERKS~NPPIRGWIICAAAFI2LEYVR
FG03177.1 	MTVATICSRGTSQEPQFIDYPKPRQG2PETTIKQRKLD~NPVLSGSILVFIAFI2INLIR
AN0828.1  	---MASSKSDASFIN----YPEIREG~PSVPYKNEDQS~IPVFRGTPLVIGASL2ISNIS
          	    :    . *        *    *  .     .  .  .   .      :   :. : 

MG11078.1 	FLREMAWHNAGFGSLRNVRRIFEDYEPLFEPTVVPLPDKEEEEMTNG----SDVPVEFAP
NCU04092.1	FIREMIWRNAGFGDLRKIRPYIEDYEPWFEPQIVPFPDFETTDEVKDDLADDEIPETEAA
FG03177.1 	FIREIVWTNAGFGSLRGIREALEVYEARYDPAVIPLPNTTAQADIES-------QQTATP
AN0828.1  	FVQSFFWRGAGFGVIREIP-NLDQWPARYDPTVIPVHIDEQS------------TSSVAN
          	*::.: * .**** :* :   :: : . ::* ::*.                      : 

MG11078.1 	QEPPTSPKQKAGRYYSAADYRALFLSGELTPTDVANAILPLIRRDTTPPGRHSVAWFNVR
NCU04092.1	KRDDLRHRYPQANYYSVEDYRKLYLSGALTPLDVAQALLPLIRRDVENPTEYSSAWFDSK
FG03177.1 	PESVIDESPSKAPYYSVADYRKLYLSGKATPTDVANALLPLIRRDINPKGKHSTAWISTR
AN0828.1  	LPVPEGRRKGKAGYYTSADYHALYQSGELTPTAVVDSLLSVTRRDTKPPGKHSVAFLECA
          	           . **:  **: *: **  **  *.:::*.: ***     .:* *::.  

MG11078.1 	VDFVLRAAEESTRRYQNKQSLGPLDGVPTAIKDEYEMDGYMTTHGSRNDYTGKDVDPETG
NCU04092.1	VDKVLAAATASTQRYKEGRSLGPLDGVPTGVKDEYDIDGYETNLGSKNIYTGTIHEEDS-
FG03177.1 	VDLVLKAAEESTIRYREGRPLGPLDGVPAAIKDDFDMDGYEMTMGSKNNYAGKAVSTGS-
AN0828.1  	VEKVRAAAEASTQRYKDGKPLGPLDGIPVTVKDEVHMDGYRRTLGTKLDFTGEFAGSTS-
          	*: *  **  ** **:: :.******:*. :**: .:***  . *::  ::*      : 

MG11078.1 	LNTNWCVRKLEEAGCVVLGKLSMHEFGLD~TTGNNPVFGTPLNPHNQGYYTGGSSSGCGY
NCU04092.1	-ITTWCVRKLQESGAIILGKLSMHEFGLD~TTGNNPVHGTPRNPHNPNYYTGGSSSGTGY
FG03177.1 	-ITNWCVGKLQDAGVIVLGKLNMHEFGMD1TTGNNPNHGTPLNPFNSKYYSGGSSSGAGY
AN0828.1  	----WCVKKWEEAGAIVIGKSTMHELGLD1TTNNNPNFGTPKNPHNPNYYCGGSSGGSGY
          	    *** * :::* :::** .***:*:* **.*** .*** **.*  ** ****.* **

MG11078.1 	AVAAGLVPFAMGSDGGGSIRIPSSLCGIVGLKPTHGRLSCLPTQNHCNTCAVLGPLAADV
NCU04092.1	AVSAGLIPIGLGSDGGGSIRIPSSLCGVFGIKPTHGRLSFKPGQNHCLTCACLGPIAADI
FG03177.1 	AVGAGLIPIALGGDAGGSIRIPSSYCSVFGLKPTHGRLSSFPTPNPAPTCCVQGPIAADM
AN0828.1  	AVGAGLVPIALGADGGGSIRIPSSFCGVWGLKPSHSRVSGFPTVGLASTVGVLGPIAASI
          	**.***:*:.:*.*.********* *.: *:**:*.*:*  *  . . *    **:**.:

MG11078.1 	RTLATAYSVVGQPHPTS------LFP-PLPPAPTLLRSPSPASPSAKTLAVPTTWFSRAS
NCU04092.1	KSLATVFQVICKPHPSS------PFP-PLKRLS--LTGPPNGGDHPKVLGIPSAWFARAT
FG03177.1 	YSLATVYDIIADPHPTS------RFP-RLYREP--------SRPLAKLLGIYEPWFDLAT
AN0828.1  	DDLALAYRIMAAPAPPSEDAKSGAFPNPLSELN---SHPAHSRPKNKTIGIMREWTDRAE
          	  ** .: ::  * *.*..:.:. **. *         .. .    * :.:   *   * 

MG11078.1 	PSVQTLCRSLMQRLVDQRGYRVVPVDLPMLTEGQIAHAIVVLTDASTLLP---DTKGLTP
NCU04092.1	PAIQTLCNTMIRRLCSDHGYTAVEIDIPFLPEGQIAHAITVLTDAATLLP---ETGNLTY
FG03177.1 	PGVQTITRRMIDKLCTEYNYEIVPITIPFTEEGQIAHAMTVLADAASLLP---ESANTTA
AN0828.1  	PAVRAVFDRALD-FYRKQNYTIIDISIPYIPEGARAHVLTIMAEIASGLSSPSQIKHLTA
          	*.::::    :  :  . .*  : : :*   **  **.:.:::: :: *.:.::    * 

MG11078.1 	ANRILLALGRTTTAV-DVLLAHKLRRMLVAHLARLWRDTLGPDALILTPTTACAGWPVRD
NCU04092.1	GNRILIALGTVTPAS-DYMLAMKLRRVLMQHLSWLWQQYPG--MLIVTPTTSCEGLPIKS
FG03177.1 	ANKLLLAMGRTTPAG-DYVLAQKLRNLLMQHLAHLWQMHPG--MMIITPTTACGGAPIRR
AN0828.1  	PNKVLVSMGMYQISGQDFLAAQRLRGLLMSHLGFLFQKHPG--LLIFTPTSPIPGWKIAG
          	 *::*:::*    : .* : * :** :*: **. *::   *   :*.***:.  *  :  

MG11078.1 	PAAELRYGLNDGDTTLASMEYVWMANFCGLPAVSVPAGFVVPEGAVGAGRPVADGEDVEG
NCU04092.1	-KSELKWGVNDGDRTLQSMEYVWLANFCGLPSISVPAGFVSPESGLVNDMDVQG----RG
FG03177.1 	GASELKHGLSDGDLTIESMTYVWLANFCGMPSLSIPAGFVVPVGQERAGEEADEN--TVG
AN0828.1  	GDADLARGVSDGKSSVRNMEYVWLANFTGCPAVSCPAGYAEDNG---------------H
          	  ::*  *:.**. :: .* ***:*** * *::* ***:.   .                

MG11078.1 	KVPVGLMAMGEWANEDGLLRFAVDAE---EVGEGLKARPPIWEDVVELARGVKGDAGREV
NCU04092.1	EVPVGLMAMGEWCAEEQLLQFGAVCE---EAGADRRRRPPIWVDVVKKAKEEVAQRG---
FG03177.1 	KVPIGLMATGEWCSEKALLEFGVEAE---AVGSDKRCKPPTWEDVLSQAKTVAQSQEKNY
AN0828.1  	RVPIGVMAMGEWGSEEDLIAFARQGEGILDLPENKEPSPKSGEQTVGLTIPRGPESVWED
          	.**:*:** ***  *. *: *.   *.      . .  *    :.:  :     .   . 

MG11078.1 	VV-------
NCU04092.1	---------
FG03177.1 	SSDSV----
AN0828.1  	VITNAQLIG
          	   . .  .
```
